# Granular straw amendment in improving soil structure and crop productivity in saline soil

**DOI:** 10.3389/fpls.2026.1887222

**Published:** 2026-07-10

**Authors:** Ting Fan, Kexin Hu, Guoshuai Wang, Delong Tian, Xudong Wang

**Affiliations:** 1 Institute of Pastoral Hydraulic Research, MWR, Hohhot, Inner Mongolia, China; 2 Yinshanbeilu Grassland Eco-Hydrology National Observation and Research Station, China Institute of Water Resources and Hydropower Research, Beijing, China; 3 College of Resources and Environment, Northwest A&F University, Yangling, Shaanxi, China

**Keywords:** biochar, granular straw, soil salinization, soil structure, wheat growth

## Abstract

Soil salinization represents a significant global threat to agricultural sustainability and the quality of arable land worldwide. This study systematically evaluated the effects of wheat straw applied in different forms—chopped (CW), granular (GW), and biochar (BW)—on soil structure and wheat growth across mild (S1), moderate (S2), and severe (S3) salinity levels in a pot experiment using saline soil from the Yellow River Basin in Inner Mongolia. Results showed that straw amendments, particularly GW and BW, markedly improved soil physical properties: GW most effectively increased the content of large macro-aggregates (>2 mm) and mean weight diameter, while BW best enhanced saturated hydraulic conductivity and the proportion of 2–0.25 mm aggregates. All straw treatments reduced soil bulk density and hardness and increased saturated water content. Compared to the no-straw control, GW, BW, and CW increased shoot biomass by 26.9%, 17.6%, and 19.3%, respectively, and significantly boosted plant nitrogen, phosphorus, and potassium uptake. Soil salinity remained a key constraint, with S1 exhibiting better aggregate stability, hydraulic conductivity, and wheat growth than S2 and S3. Path analysis identified plant potassium and nitrogen content, along with soil hydraulic conductivity and water-holding capacity, as direct drivers of biomass accumulation. In conclusion, granular straw and biochar are effective amendments for enhancing saline soil productivity by improving soil structure and mitigating salt stress, providing a viable technique for sustainable land use and grain security.

## 1 Introduction

Soil salinization represents a significant global threat to agricultural sustainability and the quality of arable land worldwide. This process induces the degradation of soil structure, which manifests as reduced aggregate stability, impaired pore architecture, diminished permeability, and lower water-holding capacity. These physical constraints collectively restrict nutrient cycling, limit organic carbon sequestration, and inhibit root development, thereby ultimately reducing land productivity (Jariani et al., 2025; Zhao et al., 2025). The Yellow River Basin in Inner Mongolia is not only a major agricultural production base but also an important ecological barrier in China. However, the widespread distribution of saline-alkali soils in this region has severely constrained agricultural productivity and ecosystem health (Yang et al., 2025). The Tumote Right Banner, located in the Hetao Plain of the Yellow River Basin, experiences intense evaporation that leads to incomplete leaching and subsequent accumulation of calcium carbonate in the lower soil profile. This process deteriorates soil physicochemical properties, severely reduces fertility, and substantially impedes crop growth and survival, ultimately resulting in diminished land productivity. Consequently, ameliorating the physical structure of saline soils to reconstruct a root-zone habitat conducive to crop growth is fundamental to restoring their productivity.

Returning agricultural waste to the fields, such as crop straw, is a widely recognized strategy for enhancing soil structure and overall quality (Li et al., 2025). Numerous studies have demonstrated that incorporating straw into soil effectively increases soil organic carbon content, promotes the formation and stabilization of soil aggregates, reduces soil bulk density and alleviates compaction, while also improving both water-holding capacity and hydraulic conductivity (Ji et al., 2024; Yang et al., 2024; Zhong et al., 2025). These enhancements in soil physical properties collectively establish a more favourable environment for crop root proliferation and development. This improved root growth environment, in turn, facilitates more efficient nutrient uptake and supports enhanced shoot biomass production (Chen et al., 2022; Wang et al., 2024). However, from an agronomic perspective, the common post-harvest practice of incorporating or spreading crop straw onto the soil surface presents operational challenges that can negatively impact the following crop cycle (Wang et al., 2020). These challenges are especially pronounced in arid and semi-arid agroecosystems, where, for example, straw residues may interfere with sowing operations by blocking machinery, induce soil drag during tillage, and physically hinder seed germination and seedling growth.

In recent years, innovative strategies for straw application have emerged, demonstrating potential advantages over traditional methods like straightforward shredding and incorporation. These novel approaches include the processing of straw into compacted granules and its thermal conversion through pyrolysis to produce biochar. For instance, straw in a granular form may provide more persistent structural benefits due to its slower decomposition and distinct physical presence within the soil matrix (Chen et al., 2023; Fan et al., 2024). Biochar, characterized by a highly porous structure, chemical stability, and abundant surface functional groups, can uniquely modify soil porosity and improve water and nutrient retention compared to raw organic materials. It can effectively modify soil porosity and structure, leading to superior retention of water and nutrients compared to untreated organic materials (Fakhar et al., 2025; Huang et al., 2024). Therefore, the physical form of returned straw may play a critical role in mediating soil amelioration outcomes and subsequent crop responses.

However, existing research has predominantly focused on the effects of straw return on non-saline or general farmland soils. A significant knowledge gap remains regarding how different physical forms of straw—specifically granules and biochar—influence soil structure formation, hydraulic properties, and crop growth under specific saline-stress conditions. The presence of salts may alter the efficacy and mechanisms of straw amendments by affecting organic matter transformation, microbial activity, and soil physio-chemical processes such as clay flocculation and pore clogging (Tang et al., 2025). However, the interactions between salinity and the distinct decomposition patterns of straw granules or the porous nature of biochar remain unclear, particularly regarding their differential effects on soil aggregation and water dynamics. Recent advances have demonstrated that straw structural modifications can improve soil pore architecture (Bai et al., 2026), enhance microbial network complexity (Zhang et al., 2026), and influence soil properties through different application strategies (Li et al., 2026) in saline-alkali soils. Nonetheless, the specific effects of granular straw on soil physical properties and crop growth under different salinity levels remain insufficiently understood. Clarifying these interactions is essential for developing targeted straw utilization technologies for saline soils.

Therefore, this study was conducted in a typical inland saline-alkali region characterized by sodic salinity. Using wheat straw, we prepared three amendment forms: chopped straw, granular straw, and biochar. Through controlled pot experiments with established salinity gradients, the study aimed to: (1) investigate the ameliorative effects of different straw forms on the composition, stability, and key hydraulic properties (e.g., bulk density, soil hardness, saturated water content, hydraulic conductivity) of saline soil; (2) determine the response patterns of wheat growth and nutrient uptake to the interaction between straw form and soil salinity; and (3) evaluate the relative contributions of improved soil physical properties versus enhanced plant nutrient status to final wheat biomass production. This work provides a mechanistic understanding and practical insights for the high-value, form-specific utilization of straw resources to achieve rapid physical improvement and productivity recovery in saline soils.

## 2 Materials and methods

### 2.1 Study site and soil characteristics

**Figure 1:**
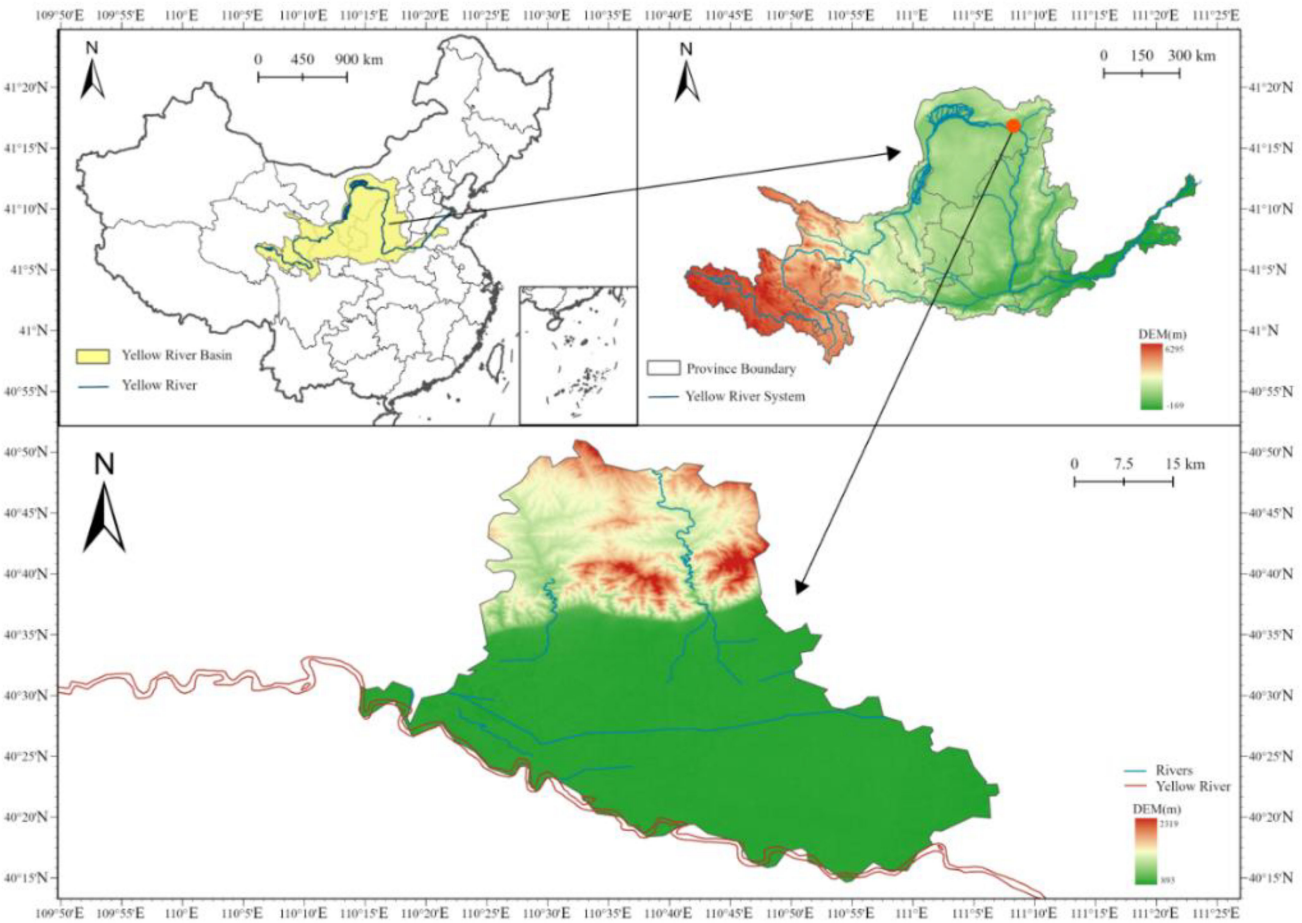
Study area.

The experimental soil was collected from Tumote Right Banner (40°33′38″N, 110°08′32″E), situated within the Yellow River Basin Irrigation District of Inner Mongolia Autonomous Region, Northwest China. The locations of the experimental site and sampling area are shown in **Figure 1**. This region lies in the Hetao Plain, a critical part of the Yellow River Basin. According to the World Reference Base for Soil Resources (WRB; ISSS/ISRIC/FAO, 1998), the soil is classified as Calcisols. This reflects the strong evaporation and low precipitation, resulting in incomplete leaching of calcium carbonate in the soil and accumulation in the lower part of the profile. The area experiences a temperate continental semi-arid to arid monsoon climate, characterized by significant temperature fluctuations, limited precipitation (mean annual: 346 mm), and exceptionally high evaporation (≥ 2100 mm). The average annual temperature is 9.0 °C, with a frost-free period of approximately 150 days. Notably, saline-alkali soils in Tumote Right Banner cover 74,000 hm², constituting 63.5% of the local arable land-a critical indicator of the severity of soil degradation in this agroecologically vulnerable region.

Calcisols are typical soils of arid and semi-arid regions, characterized by calcium carbonate accumulation, high pH, low organic matter content, and a propensity for crusting and compaction. They are widely distributed across Northwest China, Central Asia, the Mediterranean Basin, the Middle East, parts of Australia, and the southwestern United States, covering approximately 1 billion hectares globally. The findings of this study therefore have the potential to inform straw amendment practices in similar calcareous saline soils worldwide.

### 2.2 Soil sampling and pretreatment

Soil samples were collected in August 2024 from the 0–20 cm plough layer of a conventionally managed wheat-cropped field in Tumote Right Banner, Inner Mongolia Autonomous Region. The soil has been cultivated for over 30 years under a wheat–maize rotation. Following harvest of the preceding wheat crop, visible plant residues (roots, stems, and leaves) and macrofauna were manually removed to minimize organic matter heterogeneity. The soil samples were air-dried, homogenized, and sieved (<2 mm) for subsequent incubation experiments. Basic physicochemical properties of the soil (**Table 1**) were determined using standardized protocols (Pansu and Gautheyrou, 2006). Soil organic carbon (SOC): Potassium dichromate oxidation-external heating method. Total nitrogen (TN): Kjeldahl digestion–distillation method. Alkali-hydrolyzable nitrogen (AN): Alkaline diffusion method. Phosphorus fractions: Total phosphorus (TP) and available phosphorus (AP) quantified via molybdenum–antimony anti-colorimetry. Available potassium (AK): Flame photometry after ammonium acetate extraction. Particle size distribution: Pipette method with sodium hexametaphosphate dispersion. Total soluble salts: Gravimetric titration of saturation extract.

**Table 1:** The basic physical and chemical properties of the tested soil.

Physicochemical Index	Content	Salinity index	Content
Soil Organic Carbon (g kg^-1^)	7.3	pH	8.73
Total Nitrogen (g kg^-1^)	0.73	EC (μS m^-1^)	1260.54
Nitrate Nitrogen (mg kg^-1^)	23.25	K^+^ (g kg^-1^)	0.050
Ammonium Nitrogen (mg kg^-1^)	13.45	Na^+^ (g kg^-1^)	0.60
Alkali-hydro Nitrogen (mg kg^-1^)	60.23	Ca^2+^ (g kg^-1^)	0.15
Total Phosphorus (g kg^-1^)	0.63	Mg^2+^ (g kg^-1^)	0.23
Available Phosphorus (mg kg^-1^)	15.26	CO_3_^2-^ (g kg^-1^)	0.00
Available Potassium (mg kg^-1^)	162.11	HCO_3_^-^ (g kg^-1^)	0.21
Clay (<0.002 mm %)	6.62%	Cl^-^ (g kg^-1^)	0.45
Silt (0.002-0.05 mm %)	35.12%	SO_4_^2-^ (g kg^-1^)	0.82
Sand (0.05–1 mm %)	58.26%	Total salt content (g kg^-1^)	2.46

### 2.3 Soil salinity adjustment

The initial soil exhibited a salinity content of 2.46 g kg^−1^ (S1), classified as mildly saline based on established thresholds for arid agricultural soils (Shahid and Rehman, 2011). Field surveys in the study area reveal that local soil salinity varies widely, ranging from< 2.0 to > 10.0 g kg^−1^. To simulate varying salinity gradients, air-dried soil (200 g dry weight) was transferred into 500 mL culture bottles and pre-incubated with 30 mL deionized water at 25 °C for 7 days to equilibrate moisture. Salinity was then systematically elevated by adding a synthetic salt solution formulated to mirror the base cation composition (Na^+^: Ca²^+^: Mg²^+^: K^+^ = 4.3: 2.1: 1.8: 1) of the native soil. The solution contained NaHCO_3_ (8.8%), MgSO_4_ (46.9%), CaCl_2_ (13.5%), KCl (2.8%), NaCl (7.2%), and Na_2_SO_4_ (20.8%) (w/w). Incremental additions generated two salinity regimes: 4.92 g kg^−1^ (S2, moderately saline) and 7.38 g kg^−1^ (S3, severely saline). Post-adjustment, soils were re-incubated at 25 °C for 7 days under dark conditions to stabilize ionic distributions and minimize microbial activity artifacts.

### 2.4 Straw amendment preparation

Feedstock processing: Wheat straw was air-dried, partially crushed into 1–2 cm fragments, and stored in sealed plastic bags. A portion of the straw was used to produce granulated straw, while the remainder was converted to biochar.

Biochar preparation: Chopped straw was subjected to oxygen-limited pyrolysis in a muffle furnace (Yamato FO410C) preheated to 350 °C. To ensure anoxic conditions, samples were sealed in stainless steel containers under continuous N_2_ purging (1.5 L min^−1^) for 180 min. Following pyrolysis, the biochar was cooled to ambient temperature under sustained N_2_ flow, homogenized, and stored in gas-tight bags to prevent hygroscopic degradation.

Straw granulation: The dried straw was pulverized (<2 mm particle size) and mixed with a urea solution to standardize the C/N ratio to 25:1. The mixture was then extruded into cylindrical granules (8 mm diameter × 1–2 cm length) using a ring-die granulators (SKJ-650, Shandong Zhangqiu Mingchuang Machinery Co., Ltd., China). All straw amendments-chopped, granulated, and biochar-were C/N-adjusted to 25:1 to isolate physical form effects from stoichiometric variability. Key physicochemical properties of the amendments are detailed in **Table 2**. Total carbon (C) was determined via the potassium dichromate oxidation method; nitrogen (N), phosphorus (P), and potassium (K) concentrations in wheat tissues were quantified using the semi-micro Kjeldahl method, vanadate-molybdate yellow colorimetry, and flame photometry, respectively (Westerman, 1991).

**Table 2:** Basic characteristics of various forms of wheat Straw.

Straw form	pH	Bulk density (g cm^-3^)	Nutrient Content			
			C %	N %	P %	K %
Chopped wheat straw (CW)	7.2	0.19	47.5	1.9	0.1	1.7
Wheat straw Biochar (BW)	8.8	0.21	57.6	2.3	0.1	5.4
Granular wheat straw (GW)	7.3	1.06	49.6	2.0	0.1	1.6

### 2.5 Experimental design and methods

A pot experiment was conducted to investigate the effects of different straw forms on the physical properties of saline soil and wheat growth. The experiment was designed with two factors: straw form (CK: no straw; BW: biochar; CW: chopped straw; GW: granular straw) and soil salinity (S1: mild; S2: moderate; S3: severe), resulting in 12 treatments. Each treatment had 6 replicates, totaling 72 pots.

For each treatment, 2 kg of soil (sieved to<2 mm) at the designated salinity level was accurately weighed and thoroughly mixed with the corresponding straw amendment. The mixture was placed into plastic pots measuring 15 cm in diameter and 17 cm in depth with a bulk density of 1.35 g cm^−3^ after uniform packing to simulate field conditions. The application rate for chopped straw (CW) was set at 12 g kg^−1^, calculated based on the soil weight of the 20 cm plough layer. For the other forms (BW and GW), application rates were adjusted to match the carbon input of CW (12 g kg^−1^ × 47.5% = 5.70 g C kg^−1^ soil), resulting in 11.5 g kg^−1^ for GW (49.6% C) and 9.9 g kg^−1^ for BW (57.6% C). Following soil preparation, 200 mL of deionized water was added to each pot. After a 7-day pre-incubation period at room temperature, three replicates per treatment were randomly selected for planting. Ten wheat seeds were sown in each of these pots, and all pots were arranged in a completely randomized design. A further three replicates per treatment were maintained without wheat planting; these pots, which underwent all procedures except sowing, were used for measuring soil physical properties (e.g., hardness and saturated hydraulic conductivity), following all other procedures except sowing.

Throughout the cultivation period, soil moisture was maintained at 75% of field capacity by watering the pots based on weight loss. Wheat shoots were harvested at the mid-to-late jointing stage, 30 days after planting, for the determination of aboveground biomass and the analysis of nitrogen (N), phosphorus (P), and potassium (K) content. Following wheat harvest, the soil in the planted pots was subjected to a continued incubation for up to 360 days for subsequent soil aggregate analysis. Soil samples from the unplanted replicate pots were collected for the determination of soil hardness, bulk density, saturated water content, and saturated hydraulic conductivity.

### 2.6 Sample analysis and data processing

Soil hardness was determined using a digital soil hardness tester (model JC-JSD-02), with results expressed in g cm^−3^. Measurements were taken by vertically pressing the probe into the soil to a depth of 10 cm at a constant speed, with three replicates per pot averaged for each measurement. Soil bulk density and saturated water content were measured employing the core method (core volume 100 cm^3^). The core was vertically pressed into the soil, then oven-dried at 105 °C to constant weight. Bulk density was calculated as the ratio of dry soil mass to core volume, and saturated water content was determined as the mass of water retained at saturation per unit mass of dry soil. Saturated hydraulic conductivity was measured using the double-ring infiltrometer method (core volume 100 cm^3^), calculated as the water flow velocity per unit cross-sectional area perpendicular to the flow direction (mm min^−1^), using Equation 1:

(1) $$Ks=\frac{Q\times L}{S\times t\times h}$$

Where *Ks* is the saturated hydraulic conductivity; Q is the volume of water percolated through a given cross-sectional area; L is the thickness of the saturated soil layer, i.e., the distance of water flow; S is the cross-sectional area of the core; t is the time required for the percolation of water volume Q; and h is the thickness of the water layer, i.e., the hydraulic head difference.

For the analysis of soil aggregates, the wet-sieving method was applied. Specifically, 60 g of air-dried soil (<8 mm) was accurately weighed and placed on the uppermost sieve (2 mm aperture) of a nest of sieves mounted on an aggregate analyzer. The sieve series comprised apertures of 2 mm, 0.25 mm, and 0.053 mm from top to bottom. The soil sample was initially immersed in distilled water within a container for 5 minutes. Subsequently, the sieve nest was suspended on a wet-sieving apparatus and oscillated vertically in water at an amplitude of 10 cm and a frequency of 30 oscillations per minute for a duration of 5 minutes. Following sieving, the aggregates retained on each sieve were carefully washed into pre-weighed aluminium boxes. These were then oven-dried at a low temperature and weighed to determine the percentage of each aggregate size fraction. The mean weight diameter (MWD) of the aggregates was calculated using Equation 2:

(2) $$MWD=\sum_{i=1}^{n}x_{i}w_{i}/\sum_{i=1}^{n}wi$$

Where xi is the mean diameter of the i-th aggregate size fraction, and wi is the mass percentage of that fraction.

Wheat shoots were rinsed with deionized water, oven-dried at 60 °C to constant weight, and weighed to determine biomass. Total nitrogen content in plants was determined by the semi-micro Kjeldahl method. Total phosphorus content was measured by vanadate-molybdate yellow colorimetry. Total potassium content was analyzed by flame atomic absorption spectrometry.

### 2.7 Statistical analysis

Experimental data were managed and organized using Microsoft Excel 2016. A two-way analysis of variance (ANOVA) was conducted to evaluate the effects of straw form (F) and soil salinity (S) on soil physical properties (hardness, bulk density, saturated hydraulic conductivity, saturated water content, and aggregate distribution) as well as on wheat growth parameters (biomass and nutrient content). Prior to ANOVA, normality of residuals was examined using the Shapiro–Wilk test, and homogeneity of variances was verified with Bartlett test. All statistical analyses were carried out with IBM SPSS Statistics 26.0. Where significant effects were detected, Duncan new multiple range test was applied to compare means across treatment groups for each of the measured indicators. Path analysis was performed to quantify the direct and indirect linear relationships among soil physical properties, plant nutrient status, and wheat biomass. Figures and graphical representations were prepared using Origin 2023 (Origin Lab Corporation, Massachusetts, USA).

## 3 Results

### 3.1 Changes in saline soil aggregate distribution and stability under different straw forms

**Figure 2:**
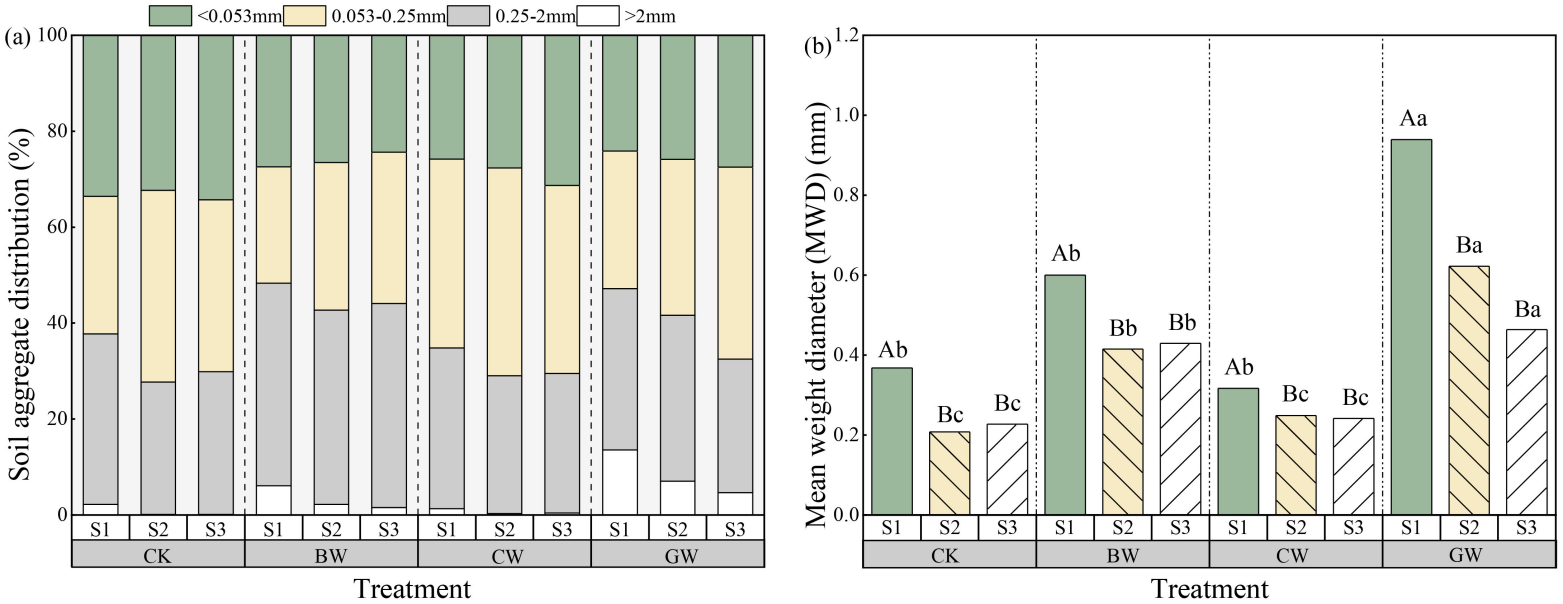
Changes in soil aggregate distribution of several particle sizes **(a)** and mean weight diameter (MWD) **(b)** of aggregates under different treatments.

The addition of straw in different forms altered the distribution of water-stable aggregates across salinity levels (**Figure 2**). Among the treatments, the content of large macro-aggregates (>2 mm) was highest in the granular straw (GW) treatment, followed by the biochar (BW) treatment. No significant difference was observed between the chopped straw (CW) and control (CK) treatments. Compared to the CW treatment, the GW and BW treatments increased the content of large macro-aggregates by 11.2-fold and 3.7-fold, respectively. The content of small macro-aggregates (2-0.25 mm) was highest in the BW treatment, being 34.8% and 30.4% higher than the CW and GW treatments, respectively. The micro-aggregate content (0.25-0.053 mm) was highest in the CW treatment, while the silt-clay fraction (<0.053 mm) was highest in the CK treatment across all treatments. Across different salinity levels, the contents of both large macro-aggregates (>2 mm) and small macro-aggregates (2-0.25 mm) were highest under the S1 salinity. Compared to S2 and S3, the S1 salinity increased the content of large macro-aggregates by 139.7% and 245.2%, and the content of small macro-aggregates by 10.1% and 12.1%, respectively.

Aggregate stability, indicated by the mean weight diameter (MWD), also varied among treatments (**Figure 2**). Both straw form and soil salinity significantly affected aggregate MWD (**Table 3**). The aggregate MWD for different straw amendment treatments followed the order: GW > BW > CW > CK. Specifically, the GW and BW treatments increased aggregate MWD by 150.8% and 78.9%, respectively, compared to the CW treatment. Aggregate MWD was highest under the S1 salinity, being significantly greater by 48.9% and 63.3% compared to S2 and S3, respectively. There was no significant difference in aggregate MWD between the S2 and S3 salinity levels. Overall, both granular straw amendment and lower salinity (S1) were most effective in enhancing aggregate size and stability.

**Table 3:** Two-way ANOVA analysis of the interaction between oil soil salinity and straw form on soil physical properties, wheat biomass and nutrient content.

Index	Soil salinity		Straw form		Soil salinity× Straw form	
	*F*	*P*	*F*	*P*	*F*	*P*
MWD of soil aggregates	51.29	< 0.001	109.28	< 0.001	7.77	< 0.001
Soil bulk density	2.43	0.109	22.76	< 0.001	0.44	0.844
Soil hardness	17.59	< 0.001	292.60	< 0.001	1.90	0.121
Soil saturation moisture content	6.62	0.005	19.13	< 0.001	0.38	0.883
Soil saturated hydraulic conductivity	59.61	< 0.001	65.95	< 0.001	5.64	0.001
Wheat plant biomass	1576.59	< 0.001	12.52	< 0.001	4.95	0.002
N content of wheat	127.50	< 0.001	1.53	0.231	0.76	0.605
P content of wheat	2608.84	< 0.001	11.54	< 0.001	3.31	0.016
K content of wheat	3417.53	< 0.001	53.18	< 0.001	17.60	< 0.001

Note: Different lowercase letters represent significant differences (*P* < 0.05) between different forms of straw at the same salinity; different capital letters represent significant differences (*P* < 0.05) between three soil salinity levels at the same form of straw application; the same below.

### 3.2 Changes in saline soil compaction and water characteristics under different straw forms

**Figure 3:**
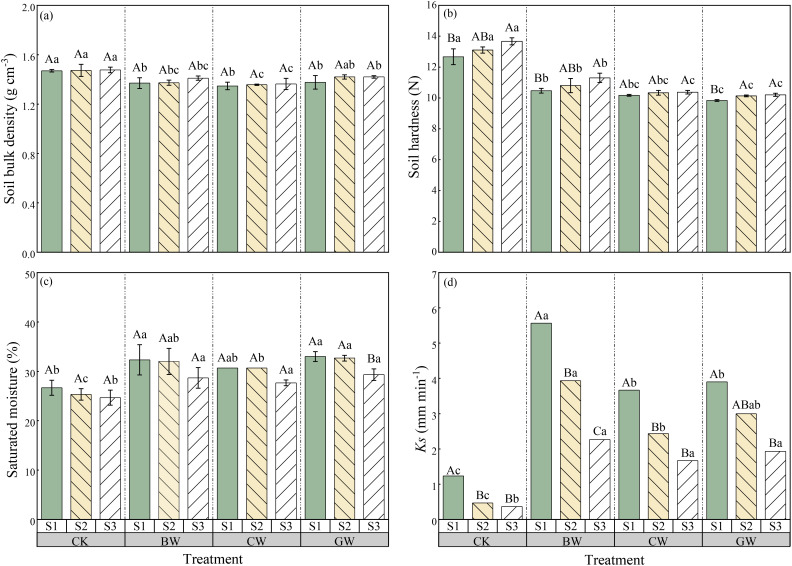
Changes in soil bulk density **(a)**, hardness **(b)**, saturated water content **(c)**, and saturated hydraulic conductivity (*K*s) **(d)**, under different treatments.

As shown in **Figure 3**, straw application altered soil bulk density, hardness, saturated water content, and saturated hydraulic conductivity across salinity levels. Straw form had a significant effect on all these soil physical properties. Soil salinity level significantly affected soil hardness, saturated water content, and saturated hydraulic conductivity but did not significantly influence soil bulk density (**Table 3**).

Straw form significantly altered soil bulk density. All straw amendments significantly reduced soil bulk density compared to CK. The CW treatment showed the lowest bulk density, though there was no significant difference among BW, CW, and GW treatments, all of which were significantly lower than CK. Compared to CK, the BW, CW, and GW treatments reduced soil bulk density by 6.0%, 8.0%, and 4.6%, respectively. There was no significant difference in soil bulk density among the three salinity levels.

Among different straw treatments, soil hardness was lowest in the GW and CW treatments, which were not significantly different from each other but were significantly lower than the BW treatment by 7.4% and 5.2%, respectively. Soil hardness was highest in the CK treatment. The BW, CW, and GW treatments significantly reduced soil hardness by 17.4%, 21.7%, and 23.5%, respectively, compared to CK. Soil hardness across salinity levels followed the order: S1< S2< S3.

Soil saturated water content also varied among treatments and was significantly influenced by both straw form and soil salinity. All three straw forms significantly increased saturated water content compared to CK. The BW and GW treatments showed no significant difference but were both significantly higher than the CW treatment. Compared to CK, the GW treatment increased saturated water content by 23.9%. Saturated water content did not differ significantly between S1 and S2 but was significantly higher in both than in S3, by 9.6% and 7.5%, respectively.

Among the straw treatments, saturated hydraulic conductivity was highest in the BW treatment, being significantly greater by 57.1% and 38.1% compared to the CW and GW treatments, respectively. There was no significant difference between CW and GW treatments, but both were significantly higher than CK, by 3.5-fold and 4.0-fold, respectively. Saturated hydraulic conductivity across salinity levels also followed the order: S1 > S2 > S3. S2 was 1.60 times higher than S3, and S1 was 1.55 times higher than S2.

### 3.3 Changes in wheat biomass and nutrient content in saline soil under different straw forms

**Figure 4:**
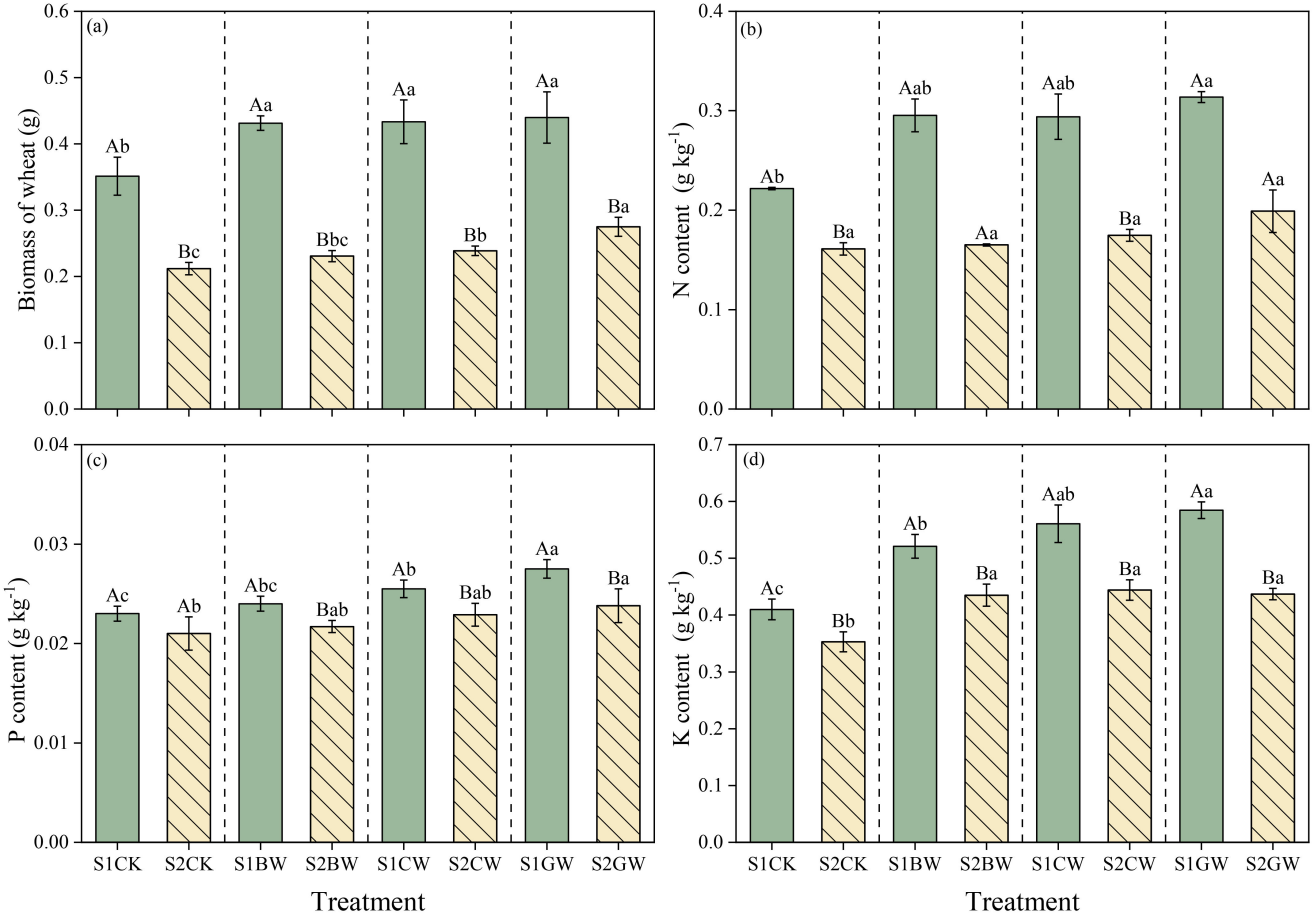
Changes in biomass **(a)** and the nutrient content of nitrogen (N) **(b)**, phosphorus (P) **(c)**, and potassium (K) **(d)** in wheat plants under different treatments.

The shoot biomass and N, P, K nutrient content of wheat plants under different treatments are shown in **Figure 4**. The S3 soil in this study was severely saline. Due to the toxic effects of salt stress, wheat seeds sown in the severely saline S3 soil failed to germinate; therefore, all wheat parameters were analyzed and compared only under the S1 and S2 salinity levels.

As shown in **Table 3**, both straw form and soil salinity significantly affected wheat shoot biomass. Compared to CK, all straw amendments significantly increased wheat shoot biomass. There was no significant difference in shoot biomass among the BW, CW, and GW treatments, but they were significantly higher than CK by 17.6%, 19.3%, and 26.9%, respectively. For each straw amendment treatment, shoot biomass under S1 salinity was significantly higher than under S2, with increases ranging from 59.9% to 86.9%.

Plant nitrogen content also varied significantly among straw treatments. Under S1 salinity, plant nitrogen content was highest in the GW treatment, being 41.5% higher than CK. Under S2 salinity, there was no significant difference in plant nitrogen content among different straw treatments. For CK and CW treatments, plant nitrogen content was significantly higher under S1 than under S2, by 37.7% and 68.3%, respectively.

Wheat plant phosphorus content was jointly influenced by straw form and soil salinity. Consequently, among the straw treatments, the GW treatment showed the highest plant phosphorus content, significantly higher than CK, BW, and CW by 16.4%, 11.8%, and 5.8%, respectively. Except for CK, plant phosphorus content under all straw amendments was higher under S1 than S2. For BW, CW, and GW treatments, plant phosphorus content under S1 was 10.6%, 11.3%, and 15.5% higher, respectively, than under S2.

Wheat plant potassium content varied between straw forms and salinity levels. All three straw forms significantly increased plant potassium content. The CW and GW treatments were not significantly different but were both significantly higher than the BW treatment, by 5.1% and 6.8%, respectively. The BW treatment increased plant potassium content by 25.3% compared to CK. Under all straw amendments, plant potassium content was higher under S1 than S2, with S1 exceeding S2 by 16.1% to 33.8%.

### 3.4 Path analysis of soil physical properties, plant nutrients, and wheat biomass

As shown in **Table 4**, the path analysis revealed distinct patterns in the factors influencing wheat biomass. Among plant nutrients, nitrogen and potassium content exerted the greatest direct effects on biomass, with direct path coefficients of 0.319 and 0.713, respectively. In contrast, plant phosphorus content had a very high total indirect effect (path coefficient = 0.858), suggesting its influence was primarily mediated through other variables rather than being direct. Regarding soil physical properties, saturated hydraulic conductivity and saturated water content showed the largest direct effects, with direct path coefficients of 0.145 and 0.125, respectively. The correlations between plant N, P, K content and wheat biomass were strong. Soil bulk density, saturated water content, and saturated hydraulic conductivity also showed relatively strong correlations with wheat biomass.

**Table 4:** Path analysis of soil physical properties, plant nutrients, and wheat biomass.

Index	Correlation coefficients	DPC	IPC								TIPC
			X1→Y	X2→Y	X3→Y	X4→Y	X5→Y	X6→Y	X7→Y	X8→Y	
X1	0.948	0.319		-0.070	0.670	0.001	0.003	-0.009	-0.053	0.085	0.629
X2	0.935	0.077	0.289		0.702	0.001	0.003	-0.007	-0.049	0.074	0.858
X3	0.959	0.713	0.300	-0.076		0.001	0.004	-0.009	-0.059	0.086	0.246
X4	0.461	0.003	0.152	-0.028	0.298		0.002	-0.015	-0.033	0.081	0.458
X5	-0.274	0.012	-0.079	0.017	-0.220	-0.001		0.022	0.092	-0.092	-0.286
X6	-0.281	0.030	-0.091	0.018	-0.221	-0.002	-0.009		0.086	-0.093	-0.311
X7	0.406	0.125	0.135	-0.030	0.339	0.001	0.009	-0.021		0.098	0.281
X8	0.622	0.145	0.188	-0.039	0.422	0.002	0.008	-0.019	-0.085	0.145	0.477

X1, N content of wheat, X2, P content of wheat, X3, K content of wheat, X4, MWD of soil aggregates, X5, Soil bulk density, X6, Soil hardness, X7, Soil saturation moisture content, X8: Soil saturated hydraulic conductivity, Y: Wheat plant biomass.

## 4 Discussion

### 4.1 Effects of straw morphology on saline soil physical properties and wheat growth

Soil structure is fundamental for healthy crop growth. Our study demonstrates that all forms of wheat straw amendment enhanced soil aggregate stability (indicated by MWD), lowered bulk density and hardness, and increased both water content at saturation and hydraulic conductivity. Specifically, straw addition increased the content of aggregates across all size classes (>2 mm, 2-0.25 mm, and 0.25-0.053 mm). This improvement is primarily attributed to the increased soil organic matter content from organic amendments, which enhances the cementation and stability of soil aggregates—a finding well-supported by previous studies (Addesso et al., 2025; Sonsri and Watanabe, 2023). These findings are consistent with the well-established understanding that organic amendments enhance soil aggregation through the supply of organic binding agents. Our results further demonstrate that this principle holds true across various forms of straw incorporation in saline soils, confirming the fundamental role of organic matter in structural remediation.

Notably, granular straw outperformed chopped straw in increasing macro-aggregate content and achieving the largest MWD. The superior performance of granular straw may be due to its expansion upon water absorption, which fosters closer contact with soil mineral particles and accelerates humus production, thereby promoting the formation of larger aggregates from micro-aggregates. This mechanism is corroborated by the work of Chen et al. (2020). Similarly, Mo et al. (2021) demonstrated that cementing substances like humus facilitate the synthesis of micro-aggregates into larger aggregates, thereby enhancing overall stability. Zhang et al. (2023) also observed that high application rates of straw granules significantly increased soil macro-aggregate content. These mechanisms collectively explain why, in our study, granular straw exhibited the highest efficiency in promoting the formation of macro-aggregates (>2 mm) and achieving the greatest MWD, as its physical expansion and subsequent humification provide ideal conditions for aggregate coalescence. However, some studies have reported conflicting results, showing that granular straw addition can reduce macro-aggregate content and MWD—a discrepancy that may be attributed to differences in application rate and soil type (Cong et al., 2020). This discrepancy with the findings of Cong et al. (2020) may be attributed to differences in application rates or soil types. Their study was conducted under different experimental conditions, suggesting that the efficacy of granular straw is context-dependent and may be optimized at appropriate application rates in saline soils. The application of biochar can promote the formation of soil macro-aggregates and improve aggregate stability, likely due to its strong adsorption capacity, which allows soil mineral particles to coalesce into larger aggregates. This is consistent with previous research (Duan et al., 2021). Sun et al. (2020) also showed that biochar return alters the particle size distribution of soil aggregates, increasing the content of large aggregates in the soil. This adsorption-mediated aggregation mechanism aligns with our observation that biochar addition significantly increased the small macro-aggregate (2-0.25 mm) content, acting as a nucleus for the binding of finer particles. In saline soils, this effect may be particularly valuable, as enhanced aggregation can mitigate salt-induced structural degradation and improve root-zone conditions.

The straw amendments also profoundly influenced key soil physical properties critical for plant growth. Soil bulk density and hardness, important indicators of soil compaction, crucial for water and nutrient movement. Among different straw treatments, granular straw application significantly reduced soil bulk density and hardness while increasing saturated water content and hydraulic conductivity. Given that saline soils are prone to crusting and compaction, which exacerbate salt stress by restricting root penetration and water movement, this reduction in hardness is particularly beneficial for creating a more favorable seedbed and root proliferation environment. This may be due to granular straw improving soil pore structure and its own strong water absorption and retention capacity (Jalota et al., 2001). It could also be because granular straw, after microbial decomposition in the soil, tends to form large pore structures, thereby enhancing soil hydraulic conductivity (Liao et al., 2024). Additionally, the porous structure and large specific surface area of biochar contributed to the observed improvements in soil hardness and water retention/conduction capacity. Previous studies have also confirmed the effects of biochar on soil bulk density and water retention (Guo et al., 2025; Zhang, et al., 2025). Collectively, these improvements in soil physical properties can be attributed to the distinct modes of action of each straw form: granular straw optimizes pore structure through its physical expansion and gradual decomposition, while biochar contributes through its inherent porosity and stable architecture. Therefore, both granular straw and biochar represent viable alternatives to conventional chopped straw for the physical amelioration of saline soils, offering targeted approaches to enhance soil quality and promote the sustainable utilization of straw resources in salt-affected regions.

Straw return can improve crop biomass and nutrient content by ameliorating soil physical structure and enhancing seedbed conditions. This study found that biochar, chopped straw, and granular straw all increased wheat shoots biomass and nutrient content. This is likely because straw application altered the root growth environment of wheat, thereby promoting its absorption of nutrients and water (Zhang et al., 2025). Liu et al. (2024) also reported that straw return significantly increased above-ground dry matter accumulation in wheat. These results corroborate previous findings that straw return, regardless of its physical form, enhances crop performance by improving the root-zone environment, thereby facilitating more efficient acquisition of water and essential nutrients. Other studies have shown that straw converted to biochar and returned to the field also significantly increased wheat biomass and nutrient content (Hu et al., 2021; Korai et al., 2021). This consistency across studies reinforces the role of biochar as an effective soil amendment for enhancing crop productivity in degraded lands. In the context of saline soils, its benefits likely extend beyond nutrient supply to include improved soil porosity and reduced salt stress through enhanced leaching. Bista et al. (2019) similarly found that biochar application promoted wheat growth and nutrient uptake, consistent with the results of this study. Notably, among different straw forms, granular straw showed the most pronounced effects. This may be because granular straw, after absorbing water and softening, integrates with the soil, improving wheat seedbed conditions. Furthermore, compared to chopped straw, the application of granular straw avoids issues such as straw covering seeds or accumulating near roots, thereby promoting wheat nutrient uptake. Shi et al. (2022) indicated that direct straw return might lead to poor seed-soil contact or uneven soil moisture, causing wheat seedling deficiency. Zhang et al. (2018) also found that granular straw return promoted wheat growth and increased seedling establishment, consistent with the findings of this study. These studies support our hypothesis that the physical integrity of granular straw mitigates the negative impacts associated with conventional chopped straw, such as poor seed-soil contact or localized nutrient immobilization. By avoiding these pitfalls, granular straw creates a more favorable micro-environment for early crop establishment and sustained nutrient uptake, explaining its superior performance in promoting wheat biomass and nutrient content observed in this study. It should be noted that this study was conducted over a single growing season with a 30-day cultivation period (up to jointing stage), and thus does not reflect the full wheat growth cycle or maturity. To evaluate the persistence of treatment effects, the soil from planted pots was incubated for an additional 360 days after harvest. Nonetheless, future studies need to verify these findings through long-term field experiments spanning multiple seasons to confirm the sustainability of the observed effects.

### 4.2 Effects of soil salinity level on saline soil physical properties and wheat growth

The formation of stable soil aggregates, crucial for protecting organic carbon, is particularly challenging in saline environments. However, their formation is inhibited in saline soils due to the prevalence of base ions, which disrupt soil structure. This study found that soil under low salinity (S1) exhibited a larger mean weight diameter (MWD) and higher aggregate stability than under higher salinity levels, which aligns with the findings of Hamoudi et al. (2013). This consistency across studies underscores the fundamental role of salinity in destabilizing soil structure, a challenge that must be addressed in saline soil remediation. Tan et al. (2024) also reported a significant negative correlation between soil aggregate water stability and salt concentration, further supporting our observation that increasing salinity progressively degraded aggregation. Consistent with these structural effects, high-salinity soil in our study showed greater hardness. This is likely because elevated base ion concentrations promote clay dispersion and reduce aggregate stability, leading to increased soil compaction and impaired water movement—a phenomenon also documented by Xing et al. (2025). Our results extend this understanding by quantifying the threshold effects across three salinity levels, demonstrating that even moderate salinity (S2) significantly compromises soil physical quality compared to mild salinity (S1).

Soil salinization is a major abiotic stress that severely limits crop growth. Our results showed that wheat shoot biomass and N, P, K nutrient contents were significantly higher in mildly saline (S1) soil than in moderately saline (S2) soil, while no seedling emergence occurred in severely saline (S3) soil due to salt stress toxicity. The failure of germination under high salinity is primarily attributed to osmotic stress and specific ion toxicity, which impede water uptake and cause metabolic dysfunction in seeds (Ibrahim, 2016; Mamedi et al., 2022). In this study, the complete failure of wheat seed germination under the S3 salinity level (7.38 g kg^−1^) reveals a critical salinity threshold beyond which the germination process of conventional seeds is completely inhibited. This indicates that severely saline soils are unsuitable for direct sowing. Therefore, reclamation measures (e.g., salt leaching, drainage improvement, or planting salt−tolerant pioneers) should precede conventional cropping. This finding further underscores the urgency and necessity of implementing active remediation measures for severely salinized soils. Furthermore, salt stress reduces wheat photosynthetic efficiency and induces long-term physiological water deficit, thereby decreasing biomass and impairing nutrient uptake (Mahboob et al., 2023; Minhas et al., 2020). Our findings corroborate these mechanisms, with the 59.9-86.9% reduction in shoot biomass from S1 to S2 demonstrating the profound impact of even moderate salinity on crop productivity.

Therefore, the pronounced negative effects of increasing salinity on both soil physical properties and wheat growth, as demonstrated in this study, highlight the critical need for effective amelioration strategies. Our findings suggest that straw-based amendments, particularly in optimized physical forms such as granules or biochar, hold promise for mitigating salt-induced degradation by improving soil structure and root-zone conditions. The superior performance of granular straw, in particular, offers a practical pathway for overcoming the limitations of conventional straw return in saline environments.

## 5 Conclusions

This study establishes that straw physical form and soil salinity are key determinants of saline soil quality and function. Technological transformation of straw—into granular form or biochar—surpassed traditional chopping in enhancing soil structure by fostering large macro-aggregates, reducing compaction, and improving hydraulic properties. These improvements translated into significantly greater wheat biomass and nutrient uptake under mild to moderate salinity. The efficacy of all amendments, however, was fundamentally limited by escalating salt stress, which ultimately prevented crop establishment in severe conditions. Mechanistically, path analysis identified plant K and N status, coupled with soil hydraulic conductivity, as direct key drivers of biomass accumulation. Therefore, employing engineered straw products like granules or biochar offers a targeted and efficient strategy for the synergistic remediation of saline soils and the enhancement of crop resilience. It should be emphasized, however, that the conclusions of this study are primarily applicable to mildly to moderately saline soils; for severely saline soils, additional desalination measures are required prior to crop establishment. This approach aligns with the principles of sustainable intensification, turning agricultural waste into a resource for building soil health and securing productivity in marginal lands.

## Data Availability

The raw data supporting the conclusions of this article will be made available by the authors, without undue reservation.
